# Salidroside Suppresses the Proliferation and Migration of Human Lung Cancer Cells through AMPK-Dependent NLRP3 Inflammasome Regulation

**DOI:** 10.1155/2021/6614574

**Published:** 2021-08-19

**Authors:** Weidong Ma, Ziyuan Wang, Yan Zhao, Qibin Wang, Yonghong Zhang, Pan Lei, Wei Lu, Shan Yan, Jun Zhou, Xiaojiao Li, Wenjun Yu, Yaoxin Zhong, Li Chen, Tao Zheng

**Affiliations:** ^1^Institute of Wudang Traditional Chinese Medicine, Taihe Hospital, Hubei University of Medicine, Shiyan, Hubei, China; ^2^Department of Pharmacy, Taihe Hospital, Hubei University of Medicine, Shiyan, Hubei, China; ^3^Hubei Key Laboratory of Wudang Local Chinese Medicine Research, Hubei University of Medicine, Shiyan, Hubei, China

## Abstract

Inflammatory reactions mediated by the NACHT, LRR, and PYD domain-containing protein 3 (NLRP3) inflammasome contributes to non-small-cell lung cancer (NSCLC) progression, particularly in patients with bacterial infections. Salidroside (SAL) has recently been shown to suppress lipopolysaccharide- (LPS-) induced NSCLC proliferation and migration, but its mechanism of action remains unclear. It has been shown that SAL improves metabolic inflammation in diabetic rodents through AMP-activated protein kinase- (AMPK-) dependent inhibition of the NLRP3 inflammasome. However, whether the NLRP3 inflammasome is regulated by SAL in NSCLC cells and how its underlying mechanism(s) can be determined require clarification. In this study, human lung alveolar basal carcinoma epithelial (A549) cells were treated with LPS, and the effects of SAL on cell proliferation, migration, AMPK activity, reactive oxygen species (ROS) production, and NLRP3 inflammasome activation were investigated. We found that LPS induction increases the proliferation and migration of A549 cells which was suppressed by SAL. Moreover, SAL protected A549 cells against LPS-induced AMPK inhibition, ROS production, and NLRP3 inflammasome activation. Blocking AMPK using Compound C almost completely suppressed the beneficial effects of SAL. In summary, these results indicate that SAL suppresses the proliferation and migration of human lung cancer cells through AMPK-dependent NLRP3 inflammasome regulation.

## 1. Introduction

Lung cancer is now the most fatal tumor globally, with estimates that by 2035, the disease will afflict more than 3 million individuals worldwide [1]. Approximately 85% of lung cancer cases are classified as non-small-cell lung cancer (NSCLC), including adenocarcinoma, squamous cell carcinoma, and large cell carcinoma [2]. Owing to the limitations in curative options, the current outcome of NSCLC is poor, with more advanced stages remaining incurable [3]. Thus, understanding the onset and development of NSCLC and finding more effective treatments are urgently needed.

Emerging evidence suggests that systemic inflammation contributes to tumorigenesis [4–6], including NSCLC [7]. In patients with lung cancer, concurrent bacterial infections enhance tumor progression [8] and increase mortality [9]. As the major pathogen in these cases, gram-negative bacteria negatively influence NSCLC through their effects on toll-like receptor- (TLR-) mediated inflammatory reactions, through the production of lipopolysaccharides (LPS) [8–10]. Moreover, the LPS-stimulated production of proinflammatory cytokines in NSCLC can predict the clinical outcome in metastatic NSCLC patients [11]. Accordingly, therapies that target LPS-induced inflammation can effectively ameliorate the adhesion and migration of NSCLC cells *in vivo* [10].

The NLRP3 inflammasome is the most well-characterized inflammatory mediator, and it is composed of NACHT, LRR, and PYD domain-containing protein 3 (NLRP3); apoptosis-associated speck-like protein containing a CARD; and caspase-1 [12]. The NLRP3 inflammasome is activated by pathogen-associated molecular patterns (PAMPs) or damage-associated molecular patterns (DAMPs), resulting in caspase-1 cleavage and the production of mature interleukin-1*β* (IL-1*β*) [6]. The NLRP3 inflammasome can also be activated by LPS under PAMPs which subsequently aggravates NSCLC [13]. Similarly, direct treatment of human lung alveolar basal carcinoma epithelial (A549) cells with IL-1*β* exhibits tumor-promoting effects *in vitro* [14].

Redox homeostasis plays an essential role in cellular functions and is also involved in the progress of cancer [15]. An imbalance in the production of reactive oxygen species (ROS) activates the NLRP3 inflammasome through either PAMPs or DAPMs. The overproduction of ROS causes the thioredoxin- (TRX-) interacting protein (TXNIP) to dissociate from TRX, inducing the NLRP3 inflammasome activation through TXNIP-NLRP3 interactions [16].

Natural products have historically made a major contribution to pharmacotherapy for cancer, and interest in natural products as drug leads provides a basis for making contributions to human health [17, 18]. Salidroside (SAL) is the main active ingredient of *Rhodiola rosea* and has been shown to exert therapeutic effects on diabetes and cardiovascular disease through its antioxidation and anti-inflammatory effects [19]. Studies suggest that SAL inhibits the growth of a range of human cancer cells including human mammary adenocarcinoma (MCF-7) [20], human mammary carcinoma (MDA-MB-231) [21], human hepatocellular carcinoma (HHCC), A549 [22], human malignant glioma (BT-325), and human gastric cancer (SGC-7901) [23]. Wang et al. recently reported that SAL decreased proliferation and induced apoptosis in A549 cells through its ability to inhibit oxidative stress and p38 [24]. In our recent study, we found that SAL improves insulin resistance in high-glucose-incubated hepatocytes through AMP-activated protein kinase- (AMPK-) mediated inhibition of the NLRP3 inflammasome [25]. However, whether the NLRP3 inflammasome is regulated by SAL in NSCLC cells remains unclear.

In this study, we investigated the effects of SAL on the LPS-induced proliferation and migration of A549 cells. We further explored the effects of SAL on ROS production and NLRP3 inflammasome activation to define its mechanism(s) of action. We herein report the ability of SAL to suppress the proliferation and migration of human lung cancer cells through AMPK-dependent NLRP3 inflammasome regulation.

## 2. Materials and Methods

### 2.1. Cell Culture

A549 cells were obtained from the China Center for Type Culture Collection (Wuhan, China). Cells were cultured in high glucose DMEM containing 10% fetal bovine serum (#04-001-1ACS, Biological Industries, Kibbutz Beit Haemek, Israel), 100 IU/ml penicillin G, and 100 *μ*g/ml streptomycin (#SV30010, HyClone, Logan, Utah, USA) at 37°C in a 5% CO_2_ atmosphere. The medium was changed every two days. Cells were digested when confluency reached 80~90%. Cell monolayers were harvested in 0.25% trypsin-EDTA solution (#25200-056, Invitrogen, Grand Island, NY, USA).

### 2.2. Cell Treatment

After culturing in 96-well plates, 6-well plates, or 35 mm dishes for 24 h, A549 cells were treated with various concentrations of LPS (#L2880, Sigma-Aldrich, St. Louis, MO, USA) or SAL (#10338-51-9, purity > 98%, Tauto Biotech, Shanghai, China) for the indicated time periods. To inhibit AMPK activity, A549 cells were cotreated with 2 *μ*M Compound C (#S7306, Selleck Chemicals, Houston, TX, USA) following treatment with LPS or SAL. Recombinant human IL-1*β* was purchased from PeproTech (#200-01B, Cranbury, NJ, USA) and added to cells at a concentration of 15 ng/ml as previously described [14].

### 2.3. Cell Viability Assay

For the assessment of cell viability, A549 cells were treated with the Cell Counting Kit-8 (CCK-8, #CK04, Dojindo Laboratories, Kumamoto, Japan) reagent according to the manufacturer's instructions.

### 2.4. Cell Migration Assay

Cell migration was examined using scratch assays as previously described [13]. Briefly, A549 cells were seeded to 100% confluence, and three scratches were introduced onto the cell layer using a sterile pipette tip. Cells were then washed in PBS three times and subsequently treated as indicated. Images were captured on a Leica DMi8 microscope (Leica Microsystems, Wetzlar, Germany) at the start of the experiment and at 24 or 48 h posttreatment. Wound healing areas were analyzed using ImageJ2x software (Wayne Rasband, National Institutes of Health, USA).

### 2.5. Oxidative Stress Measurement

A549 cells in 96-well plates were treated as described, and intracellular ROS levels were assessed using DCFH-DA (#S0033, Beyotime Institute of Biotechnology, Shanghai, China) according to the manufacturer's instructions. After loading with the probes for 20 min, plates were washed 3 times by PBS, and medium containing DCFH-DA was readded for detection. Fluorescent intensities were measured using a Tecan Infinite 200 PRO microplate reader (Tecan Group Ltd., Mannedorf, Switzerland) at excitation and emission wavelengths of 488 and 525 nm, respectively.

### 2.6. Protein Sample Preparation

Protein samples from A549 cells were extracted using a RIPA buffer (#P0013B, Beyotime Institute of Biotechnology) according to the manufacturer's instructions. Briefly, A549 cells were washed in ice-cold PBS and lysed in a RIPA buffer supplemented with a protease inhibitor cocktail (#04693132001, Roche, Basel, Switzerland) and phosphatase inhibitor cocktail (#04906845001, Roche) for 15 min. Cell lysates were collected and centrifuged at 4°C at 14,000 rpm for 15 min. Protein concentrations of the collected supernatants were determined through BCA assays (#23225, Thermo Scientific, Rockford, IL, USA). Samples were denatured in a loading buffer, boiled for 5 min, and stored at -20°C for immunoblot analysis.

### 2.7. Immunoblot Analysis

Equal amounts of proteins (~40 *μ*g) were separated on 9-11% SDS-PAGE gels and transferred to PVDF membranes. After blocking in 5% skimmed milk, membranes were probed overnight at 4°C with primary antibodies including anti-AMPK (#2532, Cell Signaling Technology, Beverly, MA, USA), anti-phospho-AMPK (#2535, Cell Signaling Technology), anti-NLRP3 (#15101, Cell Signaling Technology), anti-caspase-1 (#22915-1-AP, Proteintech, Chicago, IL, USA), anti-IL-1*β* (#sc-12742, Santa Cruz Biotechnology, Santa Cruz, CA, USA), and anti-*β*-actin (#A01010, Abbkine, Redlands, CA, USA). Membranes were washed 3 times in Tris-buffered saline with 0.1% Tween 20 and subsequently labeled with the HRP-conjugated goat anti-rabbit IgG (#A21020, Abbkine), goat anti-mouse IgG (#A21010, Abbkine), or mouse anti-Armenian hamster IgG (#sc-2789, Santa Cruz Biotechnology) at a dilution of 1 : 10000. Blots were imaged on a Tanon 5200 Chemiluminescent Imaging System (Tanon, Shanghai, China) and analyzed using ImageJ2x software.

### 2.8. Statistical Analysis

All data are expressed as the means ± SEM from at least three independent experiments. SPSS 13.0 was used for all statistical analysis. An unpaired Student *t*-test was used to compare individual groups. Multiple-group comparisons were performed using a one-way ANOVA with post hoc testing. *p* < 0.05 were considered statistically significant.

## 3. Results

### 3.1. SAL Suppresses the LPS-Induced Proliferation of A549 Cells

**Figure 1:**
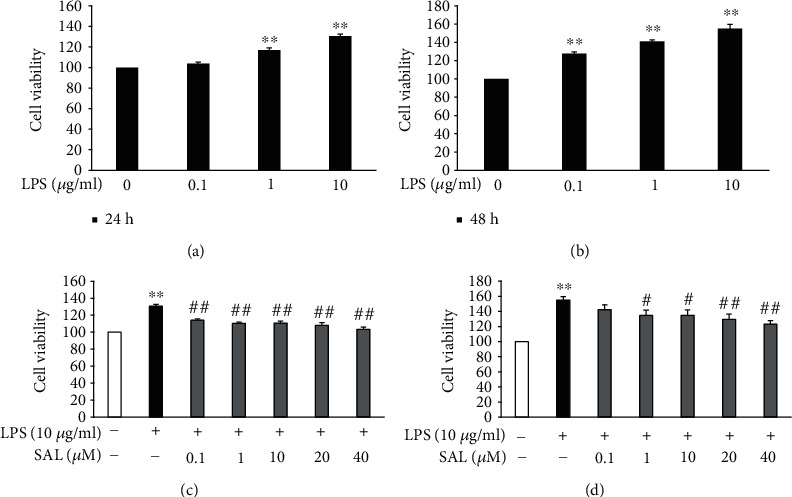
Effects of salidroside (SAL) on the proliferation of LPS-treated A549 cells. A549 cells were treated with the indicated concentrations of LPS (0.1, 1, and 10 *μ*g/ml) for either 24 h (a) or 48 h (b). Cell viabilities were then measured using CCK-8 assays. A549 cells were treated with 10 *μ*g/ml LPS in the presence or absence of SAL for either 24 h (c) or 48 h (d). Cell viabilities were then determined as described above. ^∗^*p* < 0.05 and ^∗∗^*p* < 0.01 vs. treatment without LPS; ^#^*p* < 0.05 and ^##^*p* < 0.01 vs. treatment with LPS alone. Values are means ± s.e.m. (*n* = 4).

The viability of A549 cells following their exposure to LPS (0.1, 1, and 10 *μ*g/ml) was assessed. The results showed that LPS treatment for either 24 or 48 h increased cell viability compared to vehicle controls (Figures 1(a) and 1(b)). However, coincubation with SAL (0.1, 1, 10, 20, and 40 *μ*M) for 24 and 48 h suppressed the LPS-induced increase in A549 cell proliferation in a concentration-dependent manner (Figures 1(c) and 1(d)).

### 3.2. SAL Inhibits the LPS-Induced Migration of A549 Cells

**Figure 2:**
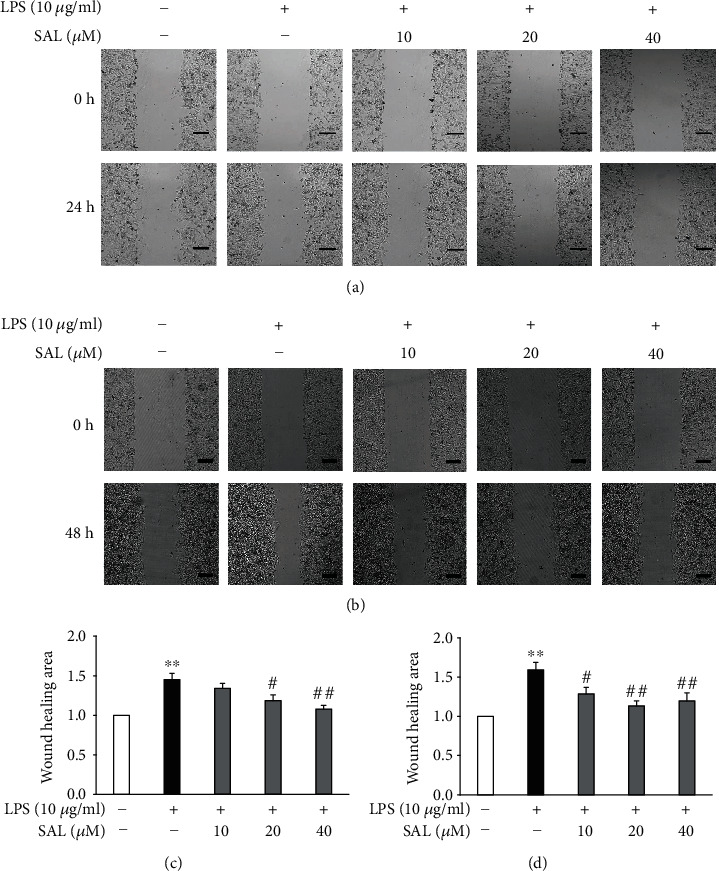
Effects of salidroside (SAL) on the migration of LPS-treated A549 cells. After exposure to 10 *μ*g/ml LPS and cotreatment with vehicle or SAL (10, 20, and 40 *μ*M) for either 24 h (a, c) or 48 h (b, d), cell migration was determined through wound healing assays. Scale bar = 200 *μ*m. ^∗^*p* < 0.05 and ^∗∗^*p* < 0.01 vs. treatment without LPS; ^#^*p* < 0.05 and ^##^*p* < 0.01 vs. treatment with LPS alone. Values are means ± s.e.m. (*n* = 5).

As shown in Figures 2(a) and 2(b), after exposure to 10 *μ*g/ml LPS for 24 or 48 h, the migration of A549 cells was markedly enhanced. In contrast, A549 cells treated with SAL at concentrations of 10, 20, and 40 *μ*M following LPS-induction showed lower levels of migration compared to cells exposed to LPS alone.

### 3.3. SAL Restores the LPS-Induced Decrease in AMPK Activity and Prevents Activation of the NLRP3 Inflammasome in A549 Cells

**Figure 3:**
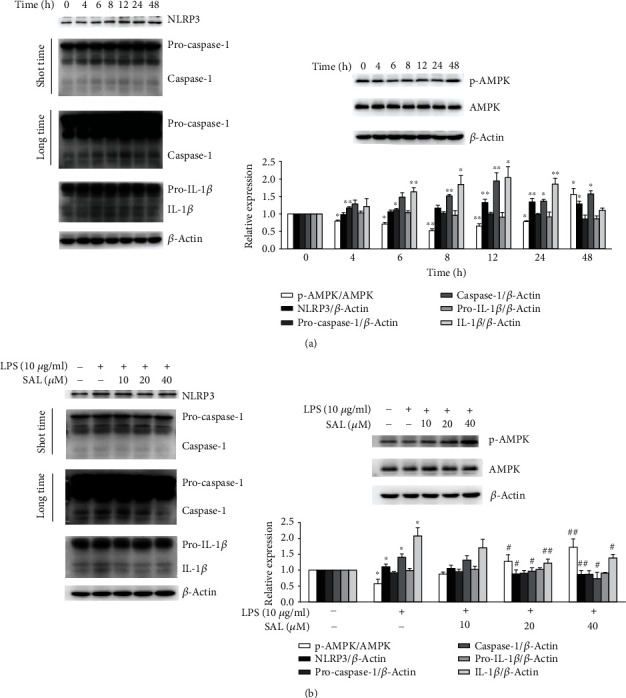
Effects of salidroside (SAL) on AMPK activity and NLRP3 inflammasome activation in A549 cells exposed to LPS. (a) A549 cells were treated with 10 *μ*g/ml LPS for 4, 6, 8, 12, 24, and 48 h, and the levels of phosphorylated-AMPK, total-AMPK, NLRP3, caspase-1, and IL-1*β* in the cell lysates were determined by immunoblotting. (b) A549 cells were cotreated with 10 *μ*g/ml LPS and various concentrations of SAL (10, 20, and 40 *μ*M) for 12 h. The levels of phosphorylated-AMPK, total-AMPK, NLRP3, caspase-1, and IL-1*β* in the cell lysates were then determined by immunoblotting. ^∗^*p* < 0.05 and ^∗∗^*p* < 0.01 vs. treatment without LPS; ^#^*p* < 0.05 and ^##^*p* < 0.01 vs. treatment with LPS alone. Values are means ± s.e.m. (*n* = 3).

Since LPS-induced NLRP3 inflammasome activation plays a critical role in the tumorigenesis of NSCLC [10, 13], we investigated the effects of SAL on the NLRP3 inflammasome in LPS-treated A549 cells. As shown in Figure 3(a), compared to untreated cells, A549 cells exposed to LPS for 4-24 h showed lower levels of AMPK phosphorylation and higher levels of NLRP3, caspase-1, and IL-1*β*. These results indicated that LPS-treated A549 cells exhibit impaired AMPK activity and enhanced NLRP3 inflammasome activation. In contrast, cells treated with SAL following LPS-induction showed higher levels of phosphorylated-AMPK. Moreover, cells treated with LPS+SAL showed significantly lower levels of NLRP3 inflammasome activation compared to those treated with LPS alone (Figure 3(b)).

### 3.4. SAL Mediates Its Effects on Cell Proliferation and Migration through the Inhibition of the NLRP3 Inflammasome

**Figure 4:**
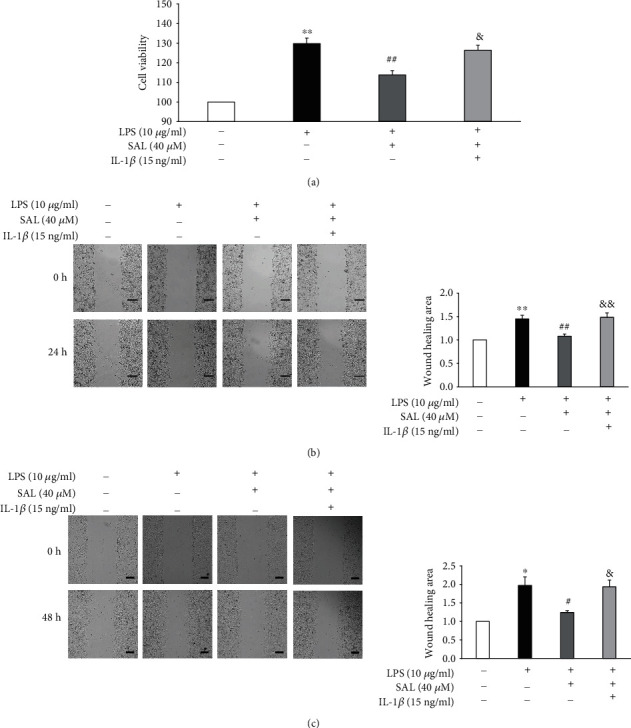
Coincubation with IL-1*β* abolishes the effects of salidroside (SAL) on cell proliferation and migration in LPS-exposed A549 cells. A549 cells were exposed to 10 *μ*g/ml LPS in the presence of SAL (40 *μ*M) and/or IL-1*β* (15 ng/ml) for 48 h. Cell viabilities were then measured using CCK-8 assays (a). After A549 cells were treated as described for 24 h (b) or 48 h (c), cell migration was determined through wound healing assays. Scale bar = 200 *μ*m. ^∗^*p* < 0.05 and ^∗∗^*p* < 0.01 vs. no LPS treatment; ^#^*p* < 0.05 and ^##^*p* < 0.01 vs. cells treated with LPS alone; ^&^*p* < 0.05 and ^&&^*p* < 0.01 vs. cells treated with LPS plus SAL. Values are means ± s.e.m. (*n* = 4).

We next investigated the role of the NLRP3 inflammasome activation in the proliferation and migration of A549 cells in response to SAL. In cells treated with either LPS or LPS+IL-1*β*, significantly higher levels of cell proliferation (Figure 4(a)) and migration (Figures 4(b) and 4(c)) were observed. These results confirmed that dysregulated NLRP3 inflammasome activation in A549 cells occurs following LPS stimulation, leading to abnormalities in cell proliferation and migration. However, the cotreatment of A549 cells with SAL suppressed the LPS-induced increase in A549 cell proliferation and migration (Figures 4(a)–4(c)). Conversely, cotreatment with IL-1*β* suppressed the effects of SAL on cell proliferation and migration in LPS-stimulated A549 cells. Taken together, these data suggest that the inhibitory effects of SAL on the NLRP3 inflammasome are necessary for its suppression on LPS-induced proliferation and migration of A549 cells.

### 3.5. SAL Decreases ROS Production and Suppresses the NLRP3 Inflammasome in an AMPK-Dependent Manner

**Figure 5:**
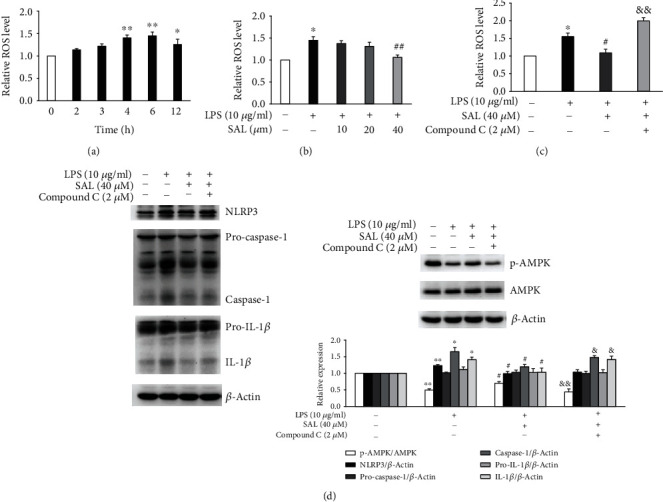
Effects of AMPK inhibition on ROS production and NLRP3 inflammasome activation in salidroside- (SAL-) treated A549 cells exposed to LPS. (a) In A549 cells exposed to 10 *μ*g/ml LPS for the indicated times, cellular ROS levels were determined using DCFH-DA. (b) In A549 cells exposed to 10 *μ*g/ml LPS in the presence of SAL at 10, 20, or 40 *μ*M for 6 h, ROS levels were measured as above. (c) In A549 cells exposed to 10 *μ*g/ml LPS in the presence of 40 *μ*M SAL or 2 *μ*M Compound C for 6 h, ROS levels were measured as described. (d) In A549 cells exposed to 10 *μ*g/ml LPS in the presence of 40 *μ*M SAL or 2 *μ*M Compound C for 12 h, the levels of phosphorylated-AMPK, total-AMPK, NLRP3, caspase-1, and IL-1*β* in the cell lysates were determined by immunoblotting. ^∗^*p* < 0.05 and ^∗∗^*p* < 0.01 vs. no LPS treatment; ^#^*p* < 0.05 and ^##^*p* < 0.01 vs. treatment with LPS alone; ^&^*p* < 0.05 and ^&&^*p* < 0.01 vs. treatment with LPS plus SAL. Values are means ± s.e.m. (*n* = 3).

Excessive ROS production is a known cause of NLRP3 inflammasome activation [6, 16]. We therefore investigated the levels of cellular ROS in LPS-treated A549 cells using DCFH-DA. As shown in Figure 5(a), ROS production increased following 2-12 h of LPS exposure. However, cotreatment with SAL at a concentration of 40 *μ*M significantly suppressed ROS generation (Figure 5(b)). SAL has been shown to relieve oxidative stress through its effects on AMPK activation [19, 25, 26]. To investigate the role of AMPK, cells were treated with the AMPK inhibitor Compound C to confirm its requirement for the regulatory activity of SAL on ROS production and the NLRP3 inflammasome activation. The results showed that the inhibition of AMPK significantly increased ROS production compared to LPS and SAL cotreated A549 cells (Figure 5(c)). In addition, Compound C treatment in LPS and SAL cotreated cells decreased the phosphorylation of AMPK and increased the levels of NLRP3, pro-caspase-1, caspase-1, pro-IL-1*β*, and IL-1*β* (Figure 5(d)). These results suggest that SAL suppresses LPS-induced activation of the ROS/NLRP3 inflammasome axis in A549 cells through its effects on AMPK.

### 3.6. AMPK Inhibition Alleviates the Effects of SAL on LPS-Induced Proliferation and Migration

**Figure 6:**
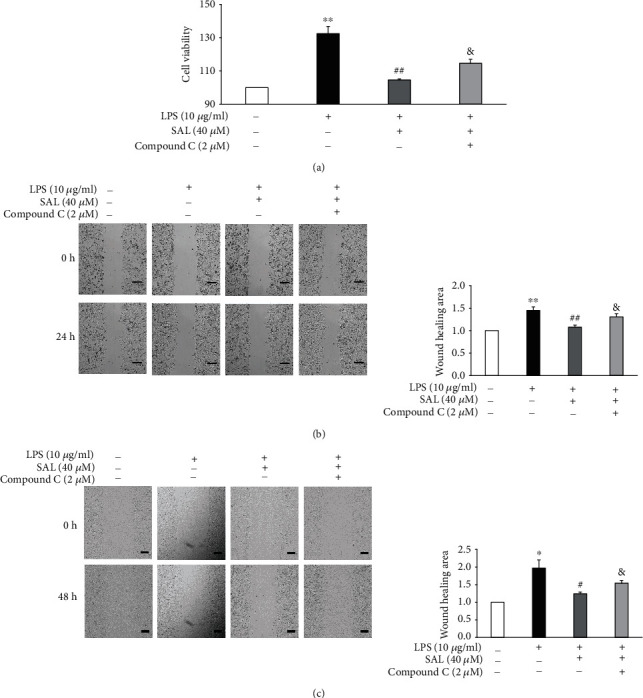
Influence of AMPK inhibition on the salidroside- (SAL-) mediated suppression of proliferation and migration in LPS-treated A549 cells. A549 cells were exposed to 10 *μ*g/ml LPS in the presence of 40 *μ*M SAL or 2 *μ*M Compound C for 48 h. Cell viabilities were then measured using CCK-8 assays (a). After A549 cells were treated as described for 24 h (b) or 48 h (c), cell migration was determined through wound healing assays. Scale bar = 200 *μ*m. ^∗^*p* < 0.05 and ^∗∗^*p* < 0.01 vs. treatment without LPS; ^#^*p* < 0.05 and ^##^*p* < 0.01 vs. treatment with LPS alone; ^&^*p* < 0.05 and ^&&^*p* < 0.01 vs. treatment with LPS plus SAL. Values are means ± s.e.m. (*n* = 3).

We next investigated the effects of AMPK inhibition on the proliferation and migration of LPS- and SAL-treated A549 cells. The inhibition of AMPK by Compound C almost completely alleviated the beneficial effects of SAL, evidenced by the decrease in both cell proliferation and migration following exposure to LPS+SAL (Figures 6(a)–6(c)). Together, these data suggest that SAL activates AMPK to suppress LPS-induced A549 cell proliferation and migration.

## 4. Discussion

**Figure 7:**
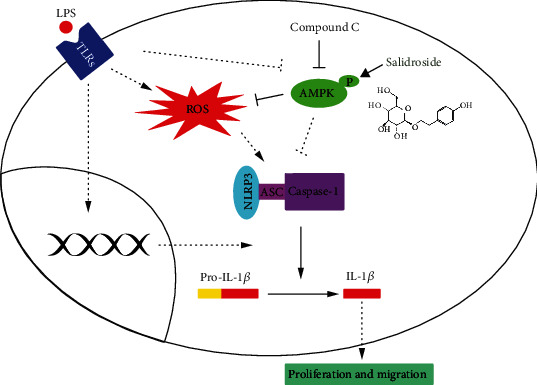
Schematic diagram highlighting the mechanism(s) of action of salidroside in NSCLC cells.

In this study, we show that the inhibitory effects of SAL on the proliferation and migration of LPS-treated A549 cells are mediated through its ability to activate AMPK and subsequently suppress the activation of the NLRP3 inflammasome. Details are summarized in Figure 7.

Inflammatory reaction contributes to tumor development and progression [27]. Accumulating evidence suggests that in NSCLC, patients with concurrent bacterial infections display more serious inflammatory reactions [9, 10]. Gram-negative bacteria are found in up to ~68% of NSCLC cases [9]. TLR4 signaling is activated by the LPS produced by these bacteria leading to inflammatory responses. Chow et al. reported that TLR4 signaling is activated following the treatment of either murine or human NSCLC cells with heat-inactivated *E. coli*, a gram-negative bacteria, and these cells also showed enhanced adhesion and migratory phenotypes [10]. It has also been reported that in NSCLC patients with gram-negative bacterial infections, the excessive activation of the TLR4/IL-33 axis promotes tumor progression [8]. In patients with metastatic NSCLC, the *ex vivo* stimulation of blood cells with LPS increased the levels of IL-6 and IL-18, which correlated to the clinical outcome of the patients [11]. These findings suggest that inflammatory signaling represents a therapeutic target for the treatment of NSCLC.

The NRLP3 inflammasome mediates the LPS-elicited inflammatory reactions that occur in response to PAMPs. Wang et al. showed that in A549 cells treated with LPS+ATP, the enhanced activation of the NLRP3 inflammasome and increased cell proliferation and migration could be reversed with siRNA targeting NLRP3 or caspase-1 inhibition [13]. These results indicated that the deregulated activation of the NLRP3 inflammasome induced by PAMPs mediates the progression of NSCLC.

The NLRP3 inflammasome contributes to the progression of a range of human cancers. For example, the activation of the NLRP3 inflammasome in macrophages that surround colorectal cancer tissue can drive cancer cell metastasis to the liver [28]. A heterozygous NLRP3 (Q705K) mutation has also been shown to be associated with a poor outcome in patients with advanced colorectal cancer [29]. Moreover, inflammatory reactions caused by the NLRP3 inflammasome in fibroblasts leads to breast cancer progression and metastasis to both the liver and lung tissue [30, 31]. Furthermore, mycoplasma hyorhinis-induced activation of the NLRP3 inflammasome has been shown to promote gastric cancer metastasis [32].

Targeting the NLPR3 inflammasome is an effective approach for cancer treatment. Zou et al. reported that polydatin can suppress the proliferation and migration of A549 and H1299 cells through inhibition of the NLRP3 inflammasome [33]. Suppressing the activation of the NLRP3 inflammasome can also prevent the outgrowth and spontaneous metastasis of triple-negative breast cancer cells [34]. Interestingly, Dumont et al. found that 5-fluorouracil- (5-FU-) induced NLRP3 inflammasome activation is a critical factor limiting its anticancer efficacy. However, the suppression of the NLRP3 inflammasome decreased 5-FU-induced IL-1*β* secretion and caspase-1 activation, enhancing its curative effects [35]. Moreover, modulation of the tumor microenvironment through the inhibition of the NLRP3 inflammasome could suppress the migration and invasion of melanoma cells [36]. GL-V9, a small-molecule AMPK activator, could prevent colitis-associated cancer through the induction of mitophagy-mediated NLRP3 inflammasome inhibition [37] or through triggering autophagy-mediated NLRP3 inflammasome degradation [38].

Accumulating evidence suggests that SAL possesses anti-inflammatory effects through its ability to inhibit the NLRP3 inflammasome. In mice with acute liver injury induced by carbon tetrachloride, treatment with SAL effectively inhibited the activation of the NLRP3 inflammasome and alleviated liver damage [39]. Similarly, SAL administration improved mechanical ventilation-induced lung injury in mice through the Sirt1-dependent inhibition of the NLRP3 inflammasome [40]. In dextran sulfate sodium-induced ulcerative colitis models, the protective effects of SAL were in part dependent on its inhibitory effects on the NLRP3 inflammasome [41]. Moreover, SAL has been shown to regulate the NLRP3 inflammasome through the TXNIP-NLRP3 pathway, providing protection against high glucose exposure, due to the accumulation of the extracellular matrix in glomerular mesangial cells [42] or through insulin resistance in hepatocytes [25]. Zhang et al. also demonstrated that SAL can alleviate Parkinson's disease through its ability to suppress pyroptosis in dopaminergic neurons, mediated by its inhibition of the NLRP3 inflammasome [43].

Wang et al. recently reported that SAL inhibits A549 cell proliferation, cell cycle progression, and metastasis and induces apoptosis through its regulatory effects on ROS generation and p38 MAPK signaling [24]. Additionally, SAL was shown to reduce the survival, migration, and invasion of A549 cells through the inhibition of Akt and MEK/ERK signaling through the upregulation of miR-195 expression [44]. In agreement with these findings, SAL could suppress the proliferation and migration of LPS-treated A549 cells. We further observed that in A549 cells treated with SAL, both the LPS-induced activation of the NLRP3 inflammasome and AMPK inhibition were effectively corrected. The inhibition of AMPK by its inhibitor Compound C almost completely alleviated the beneficial effects of SAL on A549 cell proliferation, migration, ROS production, and NLRP3 inflammasome activation. These results verify that the AMPK-signaling axis is key to the beneficial effects of SAL, not only during the pathological processes of insulin resistance and atherosclerosis [19, 45] but also during tumorigenesis. In a previous study, Wang et al. found that the expression level of epithelial-mesenchymal transition (EMT) marker snail remains unchanged in salidroside-treated A549 cells [24]. Lee et al. reported that farnesol inhibited the tumor growth of a xenograft mouse lung cancer model and abrogated the EMT process through regulating the Akt/mTOR pathway [46]. However, many previous findings have reported of entirely different actions of salidroside on Akt/mTOR signaling in human colorectal cancer cells [47] and human gastric cancer AGS cells [48]. Thus, whether EMT can be affected by salidroside and how its underlying mechanisms can be determined need to be further investigated.

Previous studies have shown that the abnormal activation of the NLRP3 inflammasome leads to an array of disease pathologies, including allergic airway disease, chronic obstructive pulmonary disease, and asbestosis [5, 49]. These findings highlight the NLRP3 inflammasome as a target for the prevention and/or treatment of lung disease. We speculate that the SAL-mediated regulation of the NLRP3 inflammasome may also improve these lung diseases, which now warrants further investigation in future studies.

In conclusion, we demonstrate that SAL suppresses the proliferation and migration of human NSCLC cells through the AMPK-dependent regulation of the NLRP3 inflammasome. This highlights the therapeutic benefits of SAL for the treatment of NSCLC, particularly in cases that are accompanied by bacterial infections.

## Data Availability

The data used to support the findings of this study are available from the corresponding author upon request.

## Abbreviations

AMPK: AMP-activated protein kinase
CCK-8: Cell Counting Kit-8
DAMPs: Damage-associated molecular patterns
EMT: Epithelial-mesenchymal transition
A549: Human lung alveolar basal carcinoma epithelial
LPS: Lipopolysaccharides
NLRP3: NACHT, LRR, and PYD domain-containing protein 3
NSCLC: Non-small-cell lung cancer
PAMPs: Pathogen-associated molecular patterns
ROS: Reactive oxygen species
SAL: Salidroside
TLR: Toll-like receptor
TRX: Thioredoxin
TXNIP: TRX-interacting protein
5-FU: 5-Fluorouracil.
